# Individualized pattern recognition for detecting mind wandering from EEG during live lectures

**DOI:** 10.1371/journal.pone.0222276

**Published:** 2019-09-12

**Authors:** Kiret Dhindsa, Anita Acai, Natalie Wagner, Dan Bosynak, Stephen Kelly, Mohit Bhandari, Brad Petrisor, Ranil R. Sonnadara

**Affiliations:** 1 Department of Surgery, McMaster University, Hamilton, Ontario, Canada; 2 Research and High-Performance Computing Support, McMaster University, Hamilton, Ontario, Canada; 3 Vector Institute for Artificial Intelligence, Toronto, Ontario, Canada; 4 Department of Psychology, Neuroscience, & Behaviour, McMaster University, Hamilton, Ontario, Canada; 5 LIVELab, McMaster University, Hamilton, Ontario, Canada; 6 Department of Health Research Methods, Evidence, and Impact, McMaster University, Hamilton, Ontario, Canada; 7 Department of Surgery, University of Toronto, Toronto, Ontario, Canada; University of Colorado Boulder, UNITED STATES

## Abstract

**Neural correlates of mind wandering:**

The ability to detect mind wandering as it occurs is an important step towards improving our understanding of this phenomenon and studying its effects on learning and performance. Current detection methods typically rely on observable behaviour in laboratory settings, which do not capture the underlying neural processes and may not translate well into real-world settings. We address both of these issues by recording electroencephalography (EEG) simultaneously from 15 participants during live lectures on research in orthopedic surgery. We performed traditional group-level analysis and found neural correlates of mind wandering during live lectures that are similar to those found in some laboratory studies, including a decrease in occipitoparietal alpha power and frontal, temporal, and occipital beta power. However, individual-level analysis of these same data revealed that patterns of brain activity associated with mind wandering were more broadly distributed and highly individualized than revealed in the group-level analysis.

**Mind wandering detection:**

To apply these findings to mind wandering detection, we used a data-driven method known as common spatial patterns to discover scalp topologies for each individual that reflects their differences in brain activity when mind wandering versus attending to lectures. This approach avoids reliance on known neural correlates primarily established through group-level statistics. Using this method for individual-level machine learning of mind wandering from EEG, we were able to achieve an average detection accuracy of 80–83%.

**Conclusions:**

Modelling mind wandering at the individual level may reveal important details about its neural correlates that are not reflected when using traditional observational and statistical methods. Using machine learning techniques for this purpose can provide new insight into the varieties of neural activity involved in mind wandering, while also enabling real-time detection of mind wandering in naturalistic settings.

## Introduction

Whether at rest or under cognitive load, the human mind is prone to turning inwards [1–4]. This spontaneous internal thinking, which can occupy up to 50% of our daily lives by some accounts [5], is often referred to as mind wandering (MW) [6, 7]. While there is evidence that MW can play a useful role in a variety of settings (e.g., for content integration, creative thinking, and future planning [8, 9]), its occurrence during attention-demanding tasks can have detrimental effects on task-related learning and performance [8, 10–13].

Studies of MW during live or recorded lectures have suggested that a greater time spent mind wandering is negatively correlated with direct educational outcomes, such as poorer retention and comprehension [14–19]. Indirect effects of MW can also occur, such as through poorer note-taking [20]. However, MW remains challenging to fully understand because we lack reliable and objective means of identifying exactly when someone is mind wandering and when they are not [7, 21]. The ability to detect MW as it occurs is, therefore, a crucial step towards improving our understanding of MW and its impacts on learning and performance. Furthermore, MW detection is an important first step towards developing approaches to counteract its negative effects. For example, real-time MW detection based on objective measures can facilitate the study of MW in a more fine-grained way and in more naturalistic settings, such as during lectures and other real-world tasks.

A major challenge in MW research is the various ways in which it has been defined throughout the literature. Many studies have either implicitly or explicitly used a content-based definition of MW, wherein any task-unrelated thought is categorized as MW [22, 23]. However, since this definition includes external stimulus-driven distractions (e.g., background noise), some researchers have opted to use a narrower definition: stimulus-independent thought [2, 9, 24]. More recently it has been argued that MW should be defined more specifically as spontaneous and unconstrained thought, which may better reflect the dynamic and uninhibited flow of thought that we internally experience when our minds wander [7]. Despite these nuances, the definition of MW varies considerably across empirical studies, and what is considered MW may partially depend on the context in which it is studied. It is therefore important to study MW in naturalistic settings not only to better understand it and its effects more fully but also to ensure that MW detection methods apply to the settings in which they are intended to be used.

### Thought probes in mind wandering research

A particular challenge in MW detection is that there is currently no known clear, objective, and externally observable indicator of MW. The most common approach to determining which time periods are likely to correspond to MW, and which are not, is to use thought probes (also known as experience sampling) [25]. Thought probes involve interrupting a given task and prompting study participants to self-report their state of attention [26, 27]. Thought probes, however, may have limited utility as a tool for MW research and detection. One reason is that thought probes artificially disrupt the task and MW alike by presenting a stimulus to cue participants to respond to the probe. In a naturalistic setting like a lecture, this method can be problematic because it requires instructor buy-in and time for student responses, which is counter to the goal of the instructor because it disrupts student learning. There is also the potential for accidental cueing of participants’ attention if lecturers are aware of when thought probes are to occur and adopt strategies to minimize disruption, such as timing lecture material in a particular way. Since thought probes are most effective when unanticipated, it is generally preferable if the lecturers are also unaware of when they will occur. In addition, the effectiveness of thought probes depends, in part, on the probe rate as well; if they are used too often, participants do not have enough time between probes to begin mind wandering again [28]. These factors make it challenging to obtain the data required to develop methods that can enable reliable continuous detection of MW, as the unreliability of self-reported MW and the likelihood that the thought probes affect the data are both factors that must be considered.

It is also important to note that some thought probes, depending on how they are presented to and interpreted by participants, may assume a content-based definition of MW [25]. However, as the neural correlates of MW are not yet fully understood, thought probes remain a useful, simple, and low-cost tool for collecting data about MW until alternative and demonstrably superior detection methods are available. Moreover, despite their limitations, thought probes may be the one the most efficient and reliable tools available with which to develop and validate potentially better methods of MW detection.

### Neural correlates of mind wandering

In recent years, neurophysiological measures, especially those acquired through functional magnetic resonance imaging (fMRI), have been increasingly used to understand the brain regions and processes that underlie MW. The default mode network (DMN), a brain network involving the posterior cingulate cortex, medial prefrontal cortex, temporoparietal cortex, and various lateral parietal regions, is often discussed in relation to MW. As its name suggests, the DMN has repeatedly been shown to be active during spontaneous thought, or when the mind is at “rest” [4, 7, 26]. More specifically, the DMN has also been shown to be active during self-reported instances of MW [8] and lapses in attention [9], and has even been shown to predict events of human error [10]. However, the DMN is also known to be activated during purposeful internal thought, including future planning and episodic memory retrieval [29–31], and is interestingly not strictly anticorrelated with the dorsal attention network [32]. This finding is important because it suggests that MW cannot simply be reduced to activation of the DMN, and activation of the DMN is not a sufficient indicator of MW.

Paradoxically, in addition to the DMN, MW also appears to be associated with activation of the executive control system of the brain, a finding that may help explain the relationship between MW and reduced task performance and learning [7, 26]. The role of the executive control network in MW remains unclear, but different hypotheses have been presented. A straightforward explanation is the control failure hypothesis, which proposes that brain regions that are a part of the executive control system attempt to reorient the brain to the task at hand during MW [33, 34]. However, there is some evidence to suggest that this may not be the case [7]. For example, direct stimulation of the nodes in the executive control network can increase task-unrelated mental activity, while the control failure hypothesis would predict a decrease of task-unrelated mental activity [35]. The decoupling hypothesis, on the other hand, proposes that the executive control network supports MW by suppressing task-related perceptual processing and orienting the brain towards personal goal-oriented thought [24, 36, 37]. However, it has been noted that this could instead reflect internal task-related thought (e.g., task-related creative problem solving), which would only be included in the broadest definitions of MW [7].

Although studies exploring functional connectivity (i.e., temporal correlations in the activity of multiple brain regions) using fMRI have contributed a wealth of information regarding the neuroanatomy and network activity related to MW, the phenomenon is thought to be a highly dynamic process that involves rapid fluctuation and spontaneity in thought. Understanding and detecting MW may, therefore, require the study of dynamic neural processes that give rise to such thinking on shorter time scales (see [12] for a review of dynamic functional connectivity with fMRI to better understand MW). EEG is a technology for acquiring neuroelectric activity with substantially greater temporal resolution (timescales in the order of a few hundred milliseconds to a few seconds versus a few seconds to few minutes with fMRI). EEG is therefore preferred for the more precise study of temporal dynamics of brain activity, albeit at the expense of the spatial resolution available with fMRI. Although EEG is largely limited to the cortex and large brain structures, it has been used to estimate the propagation of activity through less deep nodes of the neural networks established in fMRI research [38].

A primary paradigm in EEG research is the use of event-related potentials. Event-related potentials are obtained by averaging periods of recorded brain activity that are time-locked to an event or stimulus and are thus thought to reflect neural responses to that event or stimulus [39]. Analysis of the P300, an event-related potential often used as an index of cognitive processing and attention, shows a decrease in average amplitude during MW, supporting the idea that MW may reduce the amount of cognitive resources available for task-related processing [36, 40].

Researchers also use EEG to investigate the oscillatory patterns of brain activity under a variety of conditions. Measuring such oscillations requires temporally-precise sampling of the local field potentials generated by populations of neurons firing synchronously in the brain, and is thus one of the most common applications of EEG [41, 42]. Moreover, the activity in various frequency bands has been linked to a variety of cognitive functions [43–45] and states [46, 47].

To investigate MW specifically, one study measured neural oscillations with EEG in participants who were instructed to focus on their breath and press a button whenever they noticed that their attention had lapsed [40]. As expected, the authors found changes in oscillatory patterns that are associated with decreased alertness and vigilance: specifically, an increase in delta power (2–3.5 Hz; predominately in frontocentral regions) and theta power (~4–7 Hz; widespread, but most pronounced in occipital and parietocentral regions), along with decreased alpha (~9–11 Hz; focused on occipital regions) and beta power (~13–30 Hz; in frontolateral regions) during MW. Concordantly, a study investigating the relationships between oscillatory processes using a nearly identical study design found that the ratio between theta and beta activity in the frontal cortex, a measure that has been found to be negatively correlated with cognitive load [48] and attentional control [49], was higher during MW (although they did not find a relation between this measure and attentional control in their study) [50].

In a seemingly contradictory finding, another study employing thought probes while participants listened to stories discovered that not only did alpha power increase broadly over the scalp during MW (a finding that is consistent with previous work showing a similar change in alpha power associated with attentional shifts away from auditory language processing [51]), but that this change was also predictive of comprehension [52]. Together, these findings suggest that the study design and the attentive task used as a control condition may themselves elicit different subtypes (or perhaps definitions) of MW, and/or influence the neural correlates of MW that are subsequently discovered. Given these findings, there is a significant need for further translational research that aims to study MW in naturalistic settings, and for the development of objective MW detection methods that can generalize across experimental paradigms.

Given that the broader research community has not yet reached a consensus on whether MW is simply spontaneous thought, stimulus-independent thought, or more broadly, task-unrelated thought [7], the neural correlates of MW have not been fully delineated from other, potentially related, mental processes. Consequently, methods of detecting MW that are based upon already known or suspected neural correlates of MW may not fully capture the dynamics and the diversity of brain activity involved in MW, which may be especially underrepresented by current methods that discover neural correlates after aggregating responses to thought probes in group-level analyses. Moreover, which neural correlates contribute to detection may depend on the definition of MW that is most applicable for the setting. For these reasons, there is considerable interest in being able to detect MW on an online basis using objective measures as a means of furthering the study of MW in a way that more fully characterizes its dynamic and variable nature. In addition, online MW detection is relevant to education and performance science research, where the ability to monitor MW and taper the delivery of educational and training material during MW may lead to more optimal learning [17].

### Online detection of mind wandering

In the effort to better understand MW, there has been recent interest in developing methods for online detection. Previous approaches have made use of various physiological measures to detect MW, including eye tracking [53–55], heart rate variability [56, 57], and skin conductance and temperature [56, 58]. A few of these studies employed machine learning techniques to train classifiers that are, in theory, capable of automatic and continuous online detection of MW [21, 54, 55, 57, 58]. In comparison to other physiological measures, the use of neurophysiological signals for MW detection is both new and challenging. However, as MW reflects purely internal mental processes, reliable and accurate online detection of MW may ultimately necessitate the analysis of brain activity [25].

Online detection of MW with machine learning has mainly been explored with known neural correlates as provided by the cognitive neuroscience literature discussed earlier. One of the early studies demonstrating this approach used fMRI combined with measures of pupil diameter to achieve an average classification accuracy of nearly 80% [59]. While further development of this approach may enable more precise neuroimaging studies, detection of MW using fMRI is unlikely to translate well to real-world applications in naturalistic settings. Using a more portable modality for measuring the hemodynamic response in the brain to address this limitation, another group used functional near-infrared spectroscopy to detect MW [60]. However, this approach yielded a significantly reduced detection accuracy of 56% on average.

More recent work has focused on EEG for purely neurophysiological detection of MW. Combining both the event-related potential and spectral neural correlates of MW that have been discovered by previous EEG work, standard (i.e., group-level) machine learning techniques were able to achieve detection accuracies between 50% and 85%, depending on the participant [21]. Notably, the study referenced here used two different laboratory attention tasks to train a model of MW that was not overly specific to only one task.

### Individual variability in mind wandering

The overwhelming majority of previous work has focused on establishing behavioural and neural correlates of MW based on group-level analyses. This paradigm is powerful for establishing measures that are common across instances of MW and across individuals. However, individual-level analyses, especially those employing data-driven machine learning methodologies that do not rely on previously determined neural correlates of the mental process or state under investigation, have revealed surprising degrees of individual variability in the neural correlates of emotional states [61–63], the effects of concussion [64], and other areas [65, 66]. It remains an open question as to whether individual-level analysis of MW will also reveal a rich set of neural correlates that may only appear for some individuals under certain conditions.

While some have explored whether individual personality and cognitive factors contribute to MW rates [67–69], including as they relate to differences in brain activity using group-level correlations [70, 71], few studies have explored the individual variability in brain activation during MW itself. One study associated the type of self-generated thought, assessed on an individual level, to different neural correlates at the group level, demonstrating that even some degree of individual-level analysis can reveal a greater diversity of brain activation than previously discovered [6]. Here we present work in which, in addition to group-level analyses, we also use novel analytic techniques to explore brain activity on a fully individual level and perform individual-level detection of MW.

### Current study

In this study, we demonstrate, for what we believe to be the first time, machine learning-based detection of MW from EEG recorded simultaneously across the entire study sample in a naturalistic educational setting: during live lectures. Given the educational setting and the goal of identifying when participants were focused on the lecture itself, we used a general content-based definition of MW and considered a participant to be mind wandering if they were not paying attention to the lecture, as self-reported during thought probes. In addition to using a novel naturalistic setting, we employed a feature learning approach adapted from brain-computer interfacing in which patterns of brain activity associated with MW were learned on an individual basis from the data directly without constraining the models based on known neural correlates.

## Materials and methods

### Study setting and population

The study took place in the Large Interactive Virtual Environment (LIVE) Lab at McMaster University in Hamilton, Ontario, Canada [72]. This 106-seat research center and performance space allows for the measurement of brain waves using 16-channel EEG in up to 16 audience members simultaneously, serving as a unique environment for research related to the neurophysiology of music, hearing, vision, movement, and learning. Simultaneous data collection, which is a key feature of the LIVELab, ensured that all participants were exposed to the same stimuli in the same manner and accounted for the potential for students’ attention to be influenced by their peers’ behaviours, which often occurs during live lectures [73].

Upon approval from the Hamilton Integrated Research Ethics Board (HiREB-0629), all orthopedic resident trainees (N = 25) and medical students completing an elective in orthopedic surgery (N = 9) at McMaster University were invited to attend two lectures in the LIVELab. Orthopedic surgery was selected because of the program’s interest in exploring innovative teaching methods in medical education. The first lecture was given by a female, doctoral-level researcher on the topic of intimate partner violence while the second was given by a male, doctoral-level researcher on the topic of meta-analytic methods in orthopedic surgery research. Each was approximately 30 minutes in length with a 15-minute break in between.

### Procedure

We informed invitees about the study via email before the teaching session and again on the morning of the event. The study contained a behavioural component, comprised of thought probes and quizzes, and an EEG component, comprised of EEG recording during both lectures. Interested individuals could consent to participate in both the behavioural and EEG components of the study, or the behavioural component only. Assignment to these groups was determined by participants’ preference at the time of study enrollment, as some participants wished to participate in the study but preferred not to be connected to the EEG equipment. Participants provided both their verbal and written informed consent to participate in the study.

**Fig 1:**
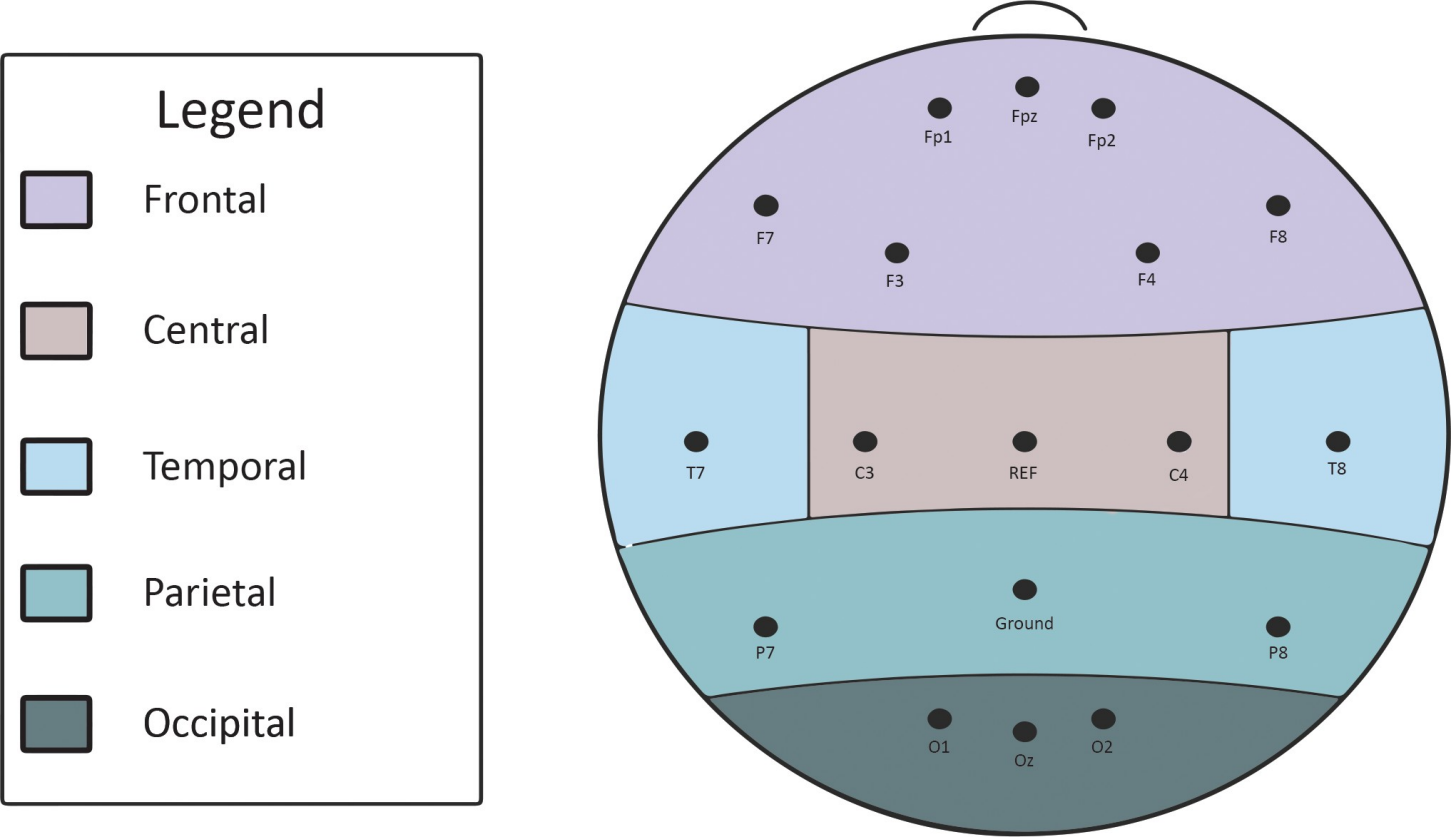
Electrode configuration for the EEG data collection.

EEG participants were fitted with caps and seated in the LIVELab. Sixteen-channel EEGs were collected simultaneously from each participant using the configuration shown in Fig 1. We interrupted each lecture approximately every four minutes with a bell ring and on-screen prompt asking participants to report their state of attention just before seeing the probe using the following question: “Just prior to seeing this probe, which of the following best describes your cognitive state?” Response options were: A) Paying attention, B) Not paying attention (i.e., mind wandering), or C) Unsure or unaware. The purpose of the probes was to identify points in the EEG data when participants self-reported that they were mind wandering versus not. Non-EEG participants also responded to the probes as a comparison group to ensure that being connected to the EEG equipment did not influence their attention.

In addition to the thought probes, we administered two quizzes after each lecture (shown in S1 and S2 Appendices). The first was administered immediately after each lecture to measure recall while the second was administered two weeks later at a teaching session to measure retention. Both quizzes contained five short answer (either fill-in-the-blank or one-word answer) questions worth one mark each that were supplied by the presenters and matched for difficulty. The questions spanned the entire lecture and were designed to test participants’ knowledge of the material covered, for example: “What is the best-reported IPV study design?” (Quiz 1, Immediate Recall) or “What term is used to describe a network with few trials and/or patients included within it?” (Quiz 2, Retention). In addition to testing content, the immediate recall quizzes also contained questions to gauge participants’ perceptions overall engagement, interest in the content of each lecture, and perceptions of presenter engagement.

### Analysis

#### Behavioural measures

We summarized behavioural data using descriptive statistics and conducted Shapiro-Wilk tests to verify assumptions of normality. Responses to the MW probes and questions about overall engagement, interest in the content, and presenter engagement were not normally distributed; thus, we opted to use non-parametric statistical tests to analyze these data. Quiz scores, on the other hand, met assumptions of normality and were therefore analyzed using parametric statistics. An exception was the retention scores for Lecture 2, which were skewed due to a floor effect caused by poor retention among participants. Since these data represented only a small portion of our data set and analyses of variance (ANOVAs) are considered relatively robust to deviations from normality [74], we opted to still use parametric analyses to analyze the quiz scores from both lectures.

We performed Mann-Whitney U tests to test for statistically significant differences in thought probe responses between EEG and non-EEG participants. We used mixed-effects ANOVAs using time of retention/recall quizzes (immediately following the lectures versus two weeks later) as the within-subjects factor and group (EEG versus non-EEG) as the between-subjects factor to test for statistically significant differences in quiz scores. We also computed Spearman’s rank-order correlation coefficients to test for associations between MW and quiz performance and used Cochran’s Q tests to determine if patterns of MW were stable over time.

The above statistical tests were conducted using IBM SPSS v. 25. The Holm-Bonferroni method of correcting for multiple comparisons was used when testing for differences between our EEG participants and behavioural-only participants (five measures).

### Neurophysiological measures

#### Preprocessing and data cleaning

We analyzed EEG data using the MNE toolbox in Python [75]. We used extensive denoising procedures because we expected greater contamination from eye movement and motor artifacts in a live lecture setting compared to traditional laboratory EEG recordings. These were performed separately for each participant. However, since some studies show that eye movements and possibly other artifacts can be indicative of MW [53–55, 76], we performed two machine learning analyses: one with artifact rejection, denoted Artifacts Suppressed, and one without artifact removal, denoted Artifacts Present. The Artifacts Suppressed approach was used to determine to what extent we could classify MW from just neuroelectric patterns and thereby promote the discovery of neural correlates of MW in naturalistic settings. In contrast, we elected to perform the Artifacts Present analysis as a comparison because in many real-world applications of MW detection, including education and performance science, the main concern is optimal detection of MW rather than reliance on purely neurological processes. In addition, since many previous MW detection studies used eye tracking rather than neural signals [53–55], the Artifacts Present approach allowed us to determine whether there was a benefit in terms of detection accuracy to skipping the use of ocular artifact suppression algorithms for real-world applications.

We removed an average of 0.53 (range: 0–2) channels per participant before analysis based on extreme variance. Remaining EEG signals were re-referenced to the average of all remaining channels and standardized using the exponential running mean and variance with a smoothness factor of 0.001, a technique for time-series standardization that reduces the influence of local fluctuations, such as high-voltage artifacts [77]. While existing research tends to associate MW with changes in EEG alpha band activity [40, 51, 52], we chose to retain a broader range of frequencies to test whether machine learning analysis would benefit from other frequency bands. Thus, we bandpass filtered the standardized EEG signals to 1–30 Hz using a Type II finite impulse response filter.

Following this simple signal preprocessing procedure, we performed two-stage artifact rejection for each participant’s EEG data. First, we epoched the entire signal into a series of non-overlapping 1s epochs. We automatically rejected epochs containing major artifacts using the *autoreject* Python toolbox [78]. This was done for both machine learning analyses (with and without independent component analysis artifact rejection), because most of the artifacts removed by *autoreject* were either of too high amplitude for the EEG amplifiers to properly characterize due to momentary loss of connection with the wireless receiver during data streaming, or due to major motion artifacts (e.g., major changes in posture).

Since *autoreject* does not remove most eye artifacts or smaller motor artifacts, we fit an independent component analysis model using the remaining epochs to separate and suppress those artifacts [79]. The *autoreject* method was used before independent component analysis because the presence of large artifacts can interfere with the technique’s ability to separate eye movements and smaller motor artifacts from the remaining EEG. Models with 16 components were fit using the extended infomax algorithm, which is well-suited to EEG data containing a variety of noise sources [80]. Since we did not directly record electrooculograms or electromyograms, we used statistical thresholding (skewness: ± 2.50, kurtosis: ± 3.00) to reject artifact-containing components and visually confirmed the selections [81]. We removed an average of 5.0 independent components (range: 3–8). We then re-mixed the signals into channel space with the artifactual components removed for further analysis.

We re-epoched the denoised EEG signals for machine learning analysis by extracting the 10s periods before each MW probe onset. Each of these 10s periods was then further epoched into five, 2s time windows and labelled according to the participant’s response to the MW probe. Epochs where the participant reported being unsure if they were MW (approximately 4% of responses), were ignored. From an initial total of 65 epochs per participant, we retained an average of 49 (range: 35–60) epochs per participant, with an average of 17 (range: 5–25) MW epochs, and 32 (range: 15–45) non-MW epochs. The exact distribution of epochs for each participant is given in Table 1 to make it easier to compare the class distribution for each participant to the subject-specific classification performance measures presented in the results.

**Table 1:** The distribution of classes across epochs and total epochs per participant.

Participant	# MW Epochs	# Non-MW Epochs	# Total Epochs
P1	25	22	47
P2	15	45	60
P3	15	45	60
P4	10	30	40
P5	5	30	35
P6	15	33	48
P7	30	30	60
P8	20	25	45
P9	10	25	35
P10	15	15	30
P11	20	40	60
P12	20	35	55
P13	15	40	55
P14	20	25	45
P15	20	35	55
**Average**	**17**	**32**	**49**

#### Statistical analysis

Since this is a novel study design in which MW was probed in a naturalistic setting and EEG was collected for the entire study group simultaneously, we performed a more traditional statistical analysis to determine whether the same neural correlates of MW reported in previous studies were also found in our study. We computed the band powers per epoch within canonical frequency bands typically used in EEG analysis (theta: 4–7 Hz, alpha: 8–12 Hz, beta1: 13–18 Hz, beta2: 19–30 Hz). To determine whether specific combinations of channels and frequency bands showed differences in band power during MW versus not MW, we fit a two-factor repeated-measures ANOVA over band powers for each channel aggregated across participants (16 total models). This included eight response variables since there are four frequency bands (theta, alpha, beta1, and beta2) over two conditions (MW versus not MW probe responses). We used probe response and frequency band as within-subject factors.

We then performed a similar analysis within each individual to determine whether MW was associated with changes in certain frequency bands. For this analysis, we fit a separate two-factor repeated-measures ANOVA over band powers for each participant aggregated across channels using probe response and frequency band as within-subject factors (15 models). We used Holm-Bonferroni corrections to correct the *p*-values across the multiple models (16 models for the channel-wise ANOVAs, and 15 models for the individual-specific ANOVAs). We report effect sizes in terms of η_*p*_^2^, where:

$$\eta_{p}^{2}=\frac{{SS}_{effect}}{{SS}_{effect}+{SS}_{error}}.$$

#### Machine learning analysis

We performed intra-subject and inter-subject classification of MW using common spatial patterns to learn discriminative spatial filters [82, 83], and a non-linear support vector machine to fit a classification model [84]. We computed six common spatial patterns (three for MW and three for not MW) and computed the log of the normalized power for each to use for classification. We used six common spatial pattern components to keep the dimensionality of the feature space low, reducing the potential for overfitting. We developed this approach as an adaptation of previous work on brain-computer interfacing [85, 86]. This approach has been developed for similar small-sample machine learning problems with EEG, where overfitting is avoided by learning a small number of features with a linear algorithm (although a greater number of samples may still lead to improved performance).

Features were learned with common spatial patterns for each of the frequency bands described earlier, and machine learning analysis was performed on each band independently. This was done so that we could determine whether the association between MW and band power changes found in prior research would remain important for single-subject detection of MW, given that the associations were discovered through the statistical analysis of EEG data averaged across participants.

Cross-validation was used to obtain statistically sound measures of classification accuracy. For each cross-validation iteration, common spatial pattern and support vector machine models were trained on a portion of the data and classification accuracy was obtained from the withheld portion of data. For intra-subject classification, we used five-fold cross-validation to partition epochs into training and test sets five different times while ensuring that epochs corresponding to the same MW probe always remained in the same set. For inter-subject classification, we used leave-one-subject-out cross-validation, meaning that each participant served as a test set once for a model trained on data aggregated from all other participants.

Due to MW occurring at a lower frequency than not MW, we report the precision, recall, and F1 score of the MW class alongside classification accuracy. The F1 score is a performance metric for binary classifiers that can be more informative for imbalanced class distributions because it considers both precision and recall as follows:

$$F1=2*\frac{precision*recall}{precision+recall}$$

Moreover, we obtained the chance-level F1 score using random permutations of the ground truth labels. We obtained a distribution of scores by randomly shuffling the ground truth labels and training our machine learning pipeline to classify the shuffled labels. This was done for 500 iterations per participant, each with a random shuffling of the ground truth labels. We then used those scores to estimate the 95% confidence interval for a chance-level F1 score. Finally, we performed independent-samples t-tests comparing these randomized permutation results with the cross-validation results that were obtained with the correct ground truth labels [87]. Chance-level F1 was computed after epoch rejection to compare classification accuracy to chance as accurately as possible.

## Results

### Participant demographics

A total of 23 individuals participated in the study. Fifteen participated in both the EEG and behavioural components and eight participated in the behavioural-only component. Further demographic information about the study participants is provided in Table 2.

**Table 2:** Participant demographics.

Demographic Variable	EEG + Behavioural (*n* = 15)	Behavioural-only (*n* = 8)
Participant type	Residents: 11 (73%) Junior: 7 (64%)^a^ Senior: 4 (36%)^a^ Medical students: 2 (13%) All in first year Research assistants: 2 (13%)	Residents: 7 (88%) Junior: 4 (57%)^a^ Senior: 3 (43%)^a^ Medical students: 1 (13%) All in first year
Age	*M* = 27.00, *SD* = 3.89	*M* = 29.50, *SD* = 2.73
Handedness	Left: 3 (20%) Right: 12 (80%)	Not applicable
Sex	Female: 3 (20%) Male: 12 (80%)	Female: 1 (13%) Male: 7 (88%)

^a^Junior residents were classified as those in years one to three of residency, while senior residents were classified as those in years four and five of residency.

### Behavioural measures

#### Mind wandering

We administered 15 thought probes across the two lectures (eight during the first and seven during the second), to which all 23 participants responded. A technical error occurred while administering the first and second probes during Lecture 1; thus, data from these probes were excluded from both the behavioural and machine learning analyses.

On average, participants reported MW during 32% of the probes in the first lecture and 38% of the probes in the second lecture, resulting in an average of 35% across both lectures. Participants were unsure about whether or not they had been MW approximately 4% of the time across both lectures. We did not find any significant differences in self-reported MW between EEG and non-EEG participants during either lecture (see Table 3 for detailed statistical results). We also did not find any significant differences in the proportion of MW at each time point for Lecture 1, *χ*^2^(5) = 6.08, *p* = 0.30; however, during Lecture 2, participants reported significantly more MW during the fourth (53% of participants) and sixth probes (56% of participants) than during the other probes (on average 16% of participants), *χ*^2^(6) = 38.67, *p* < 0.001.

**Table 3:** Comparison of EEG and non-EEG participants on various measures.

Measure	EEG + Behavioural (*n* = 15)	Behavioural-only (*n* = 8)	Statistic, Corrected *p-*value
**Lecture 1**			
Self-reported MW	*M* = 2.53, *SD* = 0.52 “A little”	*M* = 2.50, *SD* = 0.76 “A little”	*U* = 58, *p* = 1.00
Interest in content	*M* = 2.40, *SD* = 0.91 “Interesting”	*M* = 2.00, *SD* = 0.53 “Interesting”	*U* = 45, *p* = 1.00
Presenter engagement	*M* = 2.27, *SD* = 0.70 “Engaging”	*M* = 2.13, *SD* = 1.13 “Engaging”	*U* = 53, *p* = 1.00
Immediate recall	*M* = 2.57/4.00 (64%) *SD* = 1.03 (26%)	*M* = 2.50/4.00 (63%) *SD* = 1.16 (29%)	*t*(21) = 0.14, *p* = 1.00
Retention	*M* = 2.23/5.00 (45%) *SD* = 1.52 (30%)	*M* = 1.57/5.00 (31%) *SD* = 1.23 (25%)	*t*(18) = 0.93, *p* = 1.00
**Lecture 2**			
Self-reported MW	*M* = 2.40, *SD* = 0.83 “A fair amount”	*M* = 2.13, *SD* = 0.83 “A fair amount”	*U* = 48, *p* = 1.00
Interest in content	*M* = 2.33, *SD* = 1.35 “Interesting”	*M* = 2.13, *SD* = 0.99 “Interesting”	*U* = 58, *p* = 1.00
Presenter engagement	*M* = 2.47, *SD* = 0.92 “Engaging”	*M* = 2.00, *SD* = 0.76 “Engaging”	*U* = 43, *p* = 1.00
Immediate recall	*M* = 2.80/5.00 (56%) *SD* = 1.37 (27%)	*M* = 2.50/5.00 (50%) *SD* = 0.93 (19%)	*t*(21) = 0.55, *p* = 1.00
Retention	*M* = 0.81/5.00 (16%) *SD* = 1.22 (24%)	*M* = 0.07/5.00 (0%) *SD* = 0.19 (0%)	*t*(18) = 1.57, *p* = 0.65

#### Perceptions of interest and engagement

Using a five-point Likert scale where 1 = Very uninteresting and 5 = Very interesting, participants rated the content of both lectures as interesting (Lecture 1: *M* = 3.65, *SD* = 0.88; Lecture 2: *M* = 3.74, *SD* = 1.21). Using a similar scale, they also rated both presenters as engaging (Lecture 1: *M* = 3.78, *SD* = 0.85; Lecture 2: *M* = 3.70, *SD* = 0.88). We did not find any significant differences between EEG and non-EEG participants on either measure (see Table 3).

#### Immediate recall and retention

Completion rates of the immediate recall quiz and the retention quiz administered two weeks later were 100% and 87%, respectively. One question was removed from the immediate recall quiz corresponding to the first lecture due to poor wording.

Table 3 shows descriptive and test statistics for performance on the immediate recall and retention quizzes. For both lectures, mixed-effects ANOVAs showed significantly higher scores on the immediate recall quiz than the retention quiz two weeks later, Lecture 1: *F*(1,18) = 13.24, *p* < 0.01; Lecture 2: *F*(1,18) = 47.35, *p* < 0.001. There was also an effect of lecture on retention, such that participants’ retention scores were significantly higher for Lecture 1 than for Lecture 2, *F*(1,38) = 12.49, *p* = 0.001.

**Fig 2:**
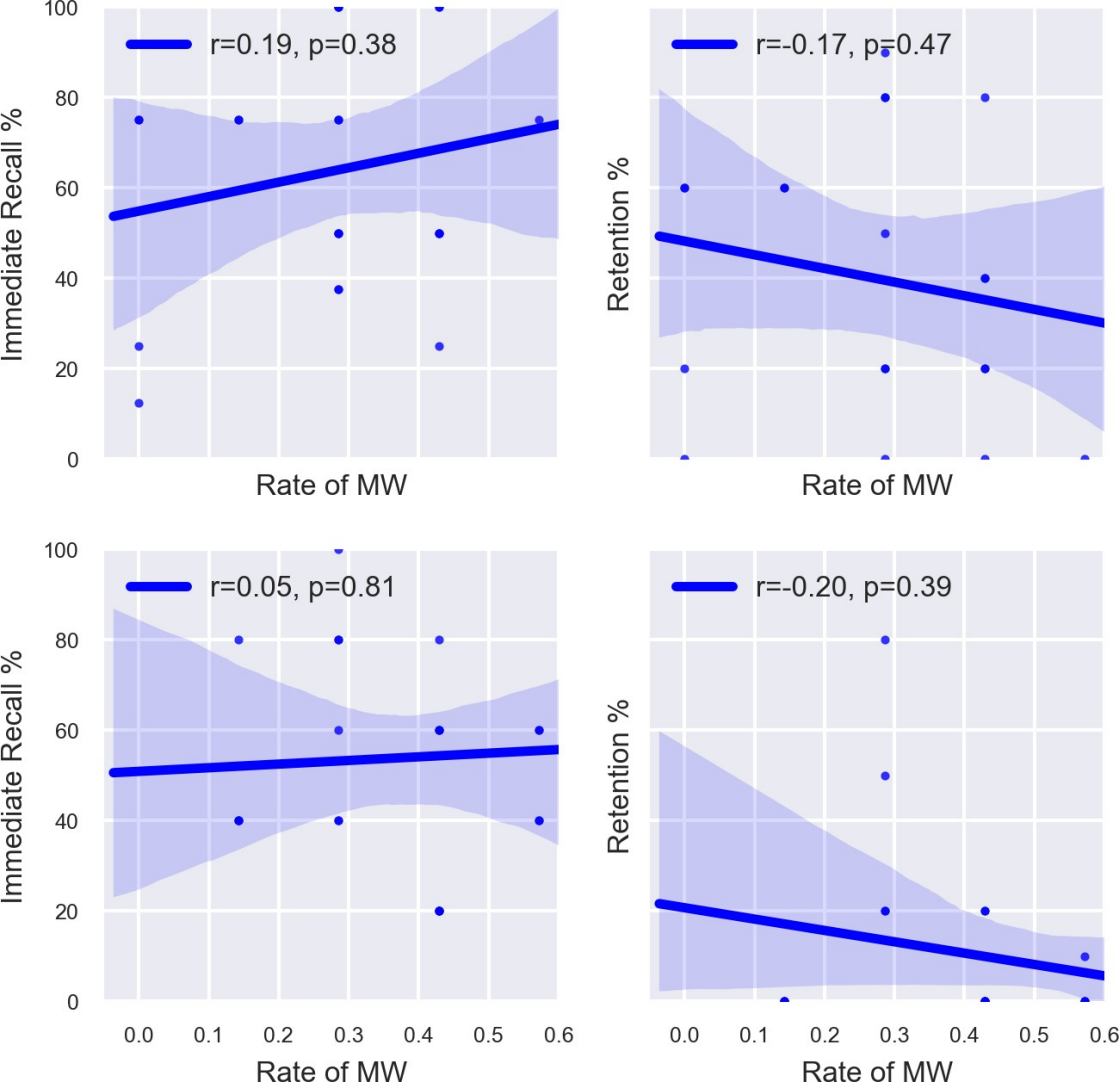
Top Left: MW rate versus immediate recall during Lecture 1. Top Right: MW rate versus retention during Lecture 1. Bottom Left: MW rate versus immediate recall during Lecture 2. Bottom Right: MW rate versus retention during Lecture 2.

We did not find any significant differences in quiz scores between EEG and non-EEG participants for either lecture (see Table 3). Moreover, the number of times MW was reported did not significantly correlate with immediate recall, Lecture 1: *r*_*S*_(23) = 0.12, *p* = 0.58; Lecture 2: *r*_*S*_(23) = -0.02, *p* = 0.94, nor retention quiz scores, Lecture 1: *r*_*S*_(20) = -0.22, *p* = 0.35; Lecture 2: *r*_*S*_(20) = -0.18, *p* = 0.45 (see Fig 2). However, we caution readers that with sample sizes of 20 and 23, these correlational analyses are considerably underpowered, with post-hoc power calculations using G*Power v. 3.1 suggesting values of 0.05–0.15.

### Neurophysiological measures

#### Power spectrum statistics

**Fig 3:**
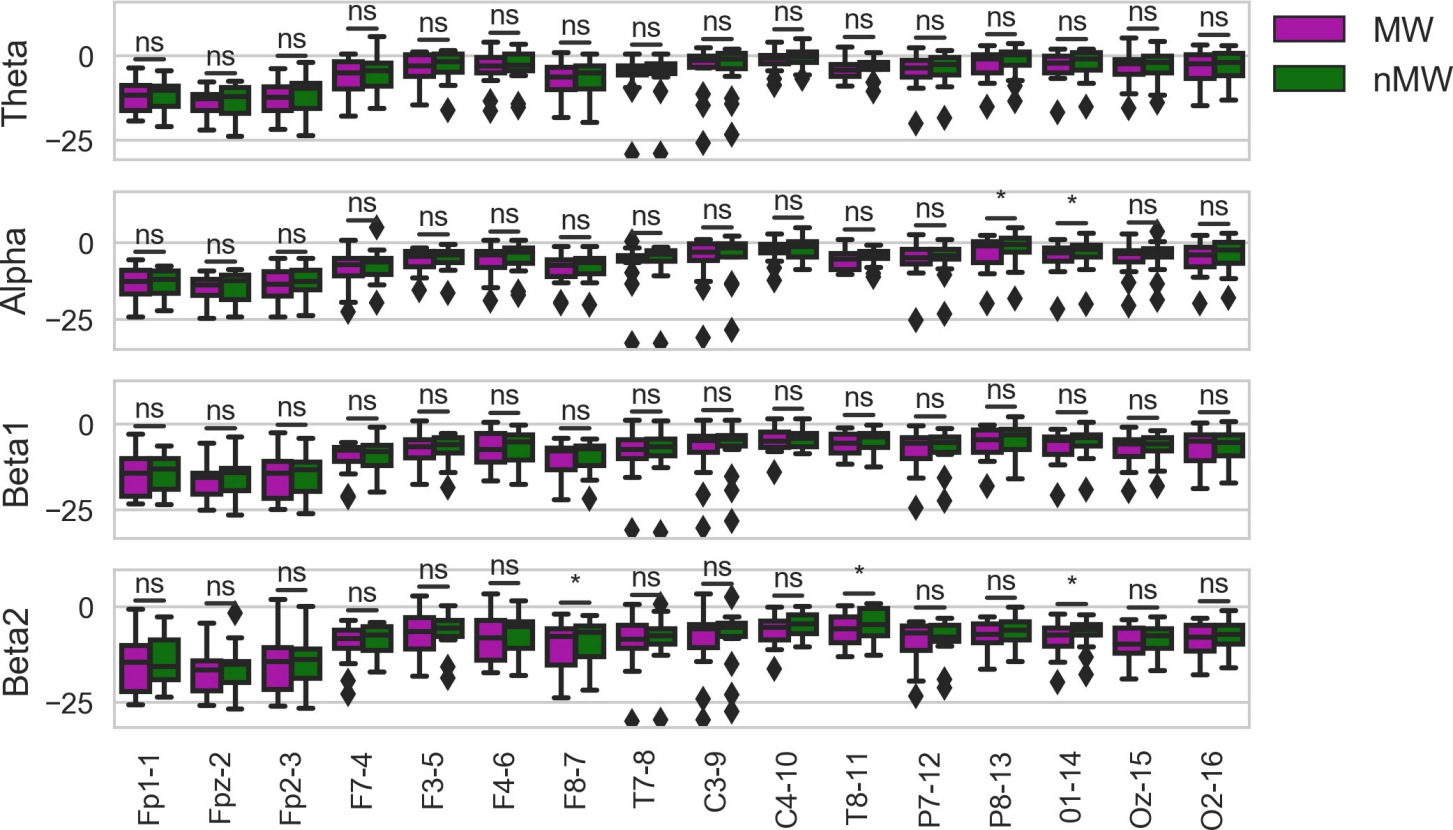
Power spectrum distributions for each channel in each frequency band, aggregated across participants. Statistical significance is calculated using paired-samples t-tests. Uncorrected *p*-values are reported as follows: *, *p* < 0.05, **, *p* < 0.01, ***, *p* < 0.001, ****, *p* < 0.0001.

The results of the channel-wise repeated-measures ANOVA models are given in Table 4. We found small to moderate effects of MW in most channels, which we would not expect to see if the neural correlates of MW were constrained to specific networks in the brain and generalizable across participants. The largest effect sizes found were in F7 (η_*p*_^2^ = 0.19, uncorrected *p* = 0.11), F8 (η_*p*_^2^ = 0.22, uncorrected *p* = 0.08), P8 (η_*p*_^2^ = 0.26, uncorrected *p* < 0.05), and O1 (η_*p*_^2^ = 0.20, uncorrected *p* = 0.08). When examining the interaction between MW and frequency band, we found only moderate effects in F8 (η_*p*_^2^ = 0.17, uncorrected *p* = 0.06), T8 (η_*p*_^2^ = 0.29, uncorrected *p* < 0.01), O1 (η_*p*_^2^ = 0.19, uncorrected *p* = 0.03), and Oz (η_*p*_^2^ = 0.16, uncorrected *p* = 0.06), possibly suggesting that the frequency bands associated with MW were not consistent across individuals. However, none of the group-level statistics were significant at a 0.05 level after correcting for multiple comparisons. Post-hoc analyses revealed a similar pattern of findings (see Fig 3), but more clearly showed that the effect of MW remaining after aggregating data from the entire group of participants may depend on the frequency band as well.

**Table 4:** Results of repeated-measures ANOVA models for each channel.

Channel	Factor	Result	Corrected *p*-value	η_*p*_^2^
Fp1	MW	*F*(1,14) = 1.98	1.0	0.12
	MW x Freq.	*F*(3,42) = 0.43	1.0	0.03
Fpz	MW	*F*(1,13) = 1.82	1.0	0.12
	MW x Freq.	*F*(3,39) = 1.35	1.0	0.09
Fp2	MW	*F*(1,13) = 2.32	1.0	0.15
	MW x Freq.	*F*(3,39) = 0.41	1.0	0.04
F7	MW	*F*(1,13) = 2.99	1.0	0.19
	MW x Freq.	*F*(3,39) = 0.57	1.0	0.04
F3	MW	*F*(1,14) = 1.17	0.87	0.08
	MW x Freq.	*F*(3,42) = 0.60	1.0	0.04
F4	MW	*F*(1,14) = 1.29	1.0	0.09
	MW x Freq.	*F*(3,42) = 0.34	1.0	0.02
F8	MW	*F*(1,13) = 3.71	1.0	0.22
	MW x Freq.	*F*(3,39) = 2.63	0.82	0.17
T7	MW	*F*(1,14) = 2.49	1.0	0.15
	MW x Freq.	*F*(3,42) = 0.41	1.0	0.03
C3	MW	*F*(1,14) = 2.02	1.0	0.13
	MW x Freq.	*F*(3,42) = 0.33	1.0	0.02
C4	MW	*F*(1,13) = 1.39	1.0	0.10
	MW x Freq.	*F*(3,39) = 0.81	1.0	0.06
T8	MW	*F*(1,12) = 2.80	1.0	0.19
	MW x Freq.	*F*(3,36) = 4.80	0.10	0.29
P7	MW	*F*(1,13) = 0.95	0.60	0.07
	MW x Freq.	*F*(3,39) = 0.36	1.0	0.03
P8	MW	*F*(1,14) = 4.80	0.73	0.26
	MW x Freq.	*F*(3,42) = 0.80	1.0	0.05
O1	MW	*F*(1,14) = 3.52	1.0	0.20
	MW x Freq.	*F*(3,42) = 3.37	0.41	0.19
Oz	MW	*F*(1,14) = 1.20	1.0	0.08
	MW x Freq.	*F*(3,42) = 2.70	0.81	0.16
O2	MW	*F*(1,14) = 1.43	1.0	0.09
	MW x Freq.	*F*(3,42) = 1.79	1.0	0.11

**Fig 4:**
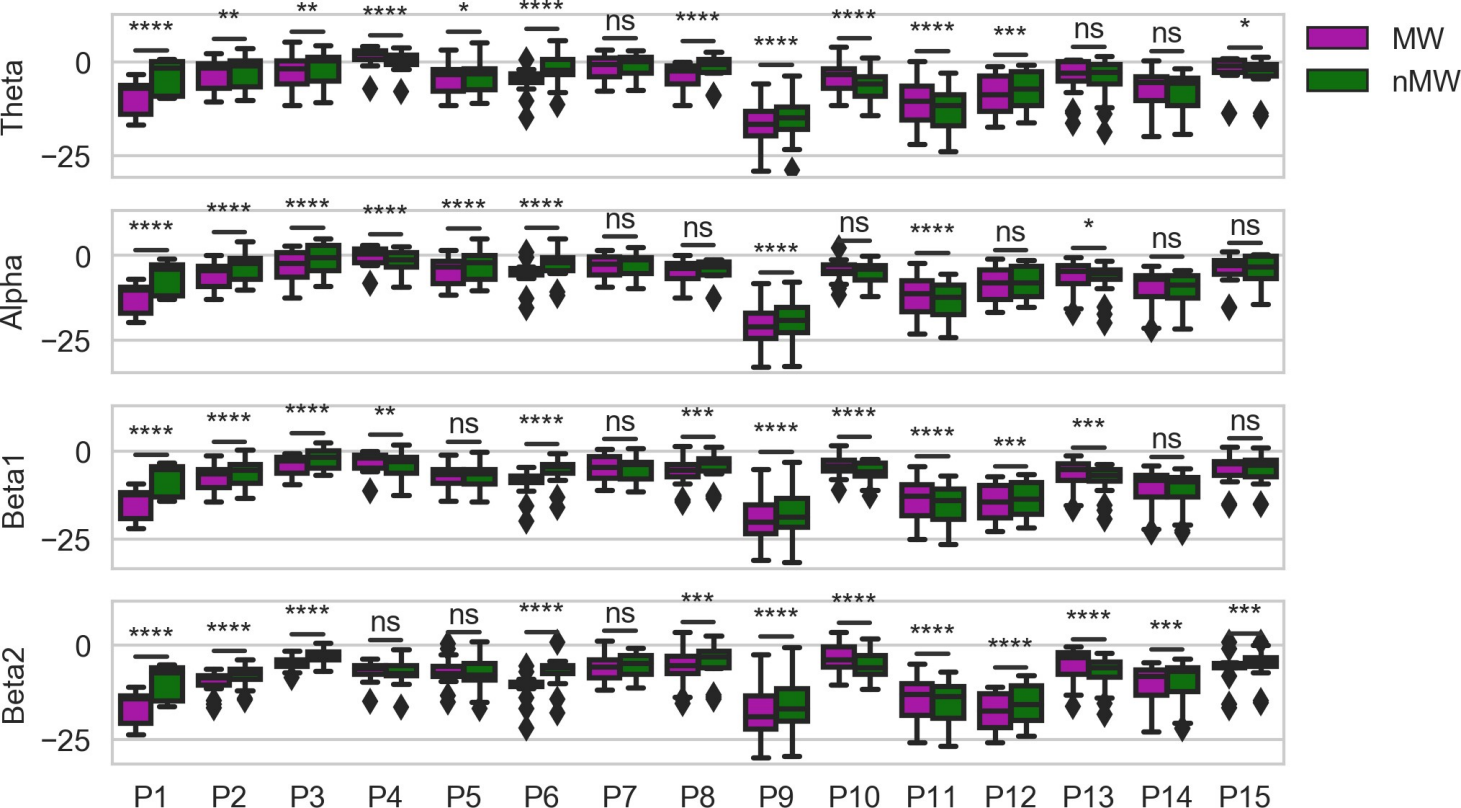
Power spectrum distributions for each participant in each frequency band, aggregated across channels. Statistical significance is calculated using paired-samples t-tests. Uncorrected *p*-values are reported as follows: *, *p* < 0.05, **, *p* < 0.01, ***, *p* < 0.001, ****, *p* < 0.0001.

The results of the individual-specific repeated-measures ANOVA models are given in Table 5. Here we found strong effects of MW and the interaction of MW and frequency band for all participants except for P7. For P14 and P15, we only found an effect of the interaction between MW and frequency band, suggesting that the effect of MW may be more isolated to a specific frequency band in those participants. Post-hoc analyses (see Fig 4) showed that the effect of MW appeared to be spread across frequency bands for most individuals, except for those individuals previously identified. For P7, we found no effect in any frequency band, and for P14 and P15, the strongest effect appeared in the beta frequencies. For some participants, MW was associated with a reduction in band power, while for others, the opposite was true.

**Table 5:** Results of repeated-measures ANOVA models for each participant.

Participant	Factor	Result	Corrected *p*-value	η_*p*_^2^
P1	MW	*F*(1,15) = 400.95	< 0.0001	0.96
	MW x Freq.	*F*(3,45) = 17.58	< 0.0001	0.54
P2	MW	*F*(1,14) = 95.49	< 0.0001	0.87
	MW x Freq.	*F*(3,42) = 38.83	< 0.0001	0.74
P3	MW	*F*(1,14) = 58.84	< 0.0001	0.81
	MW x Freq.	*F*(3,42) = 12.04	< 0.0001	0.46
P4	MW	*F*(1,13) = 21.17	0.003	0.62
	MW x Freq.	*F*(3,39) = 3.29	0.061	0.20
P5	MW	*F*(1,15) = 5.99	0.109	0.29
	MW x Freq.	*F*(3,45) = 12.86	< 0.0001	0.46
P6	MW	*F*(1,15) = 248.06	< 0.0001	0.94
	MW x Freq.	*F*(3,45) = 14.14	< 0.0001	0.49
P7	MW	*F*(1,13) = 0.88	1.0	0.06
	MW x Freq.	*F*(3,39) = 1.36	0.269	0.09
P8	MW	*F*(1,15) = 22.51	0.002	0.60
	MW x Freq.	*F*(3,45) = 24.11	< 0.0001	0.62
P9	MW	*F*(1,15) = 82.16	< 0.0001	0.85
	MW x Freq.	*F*(3,45) = 6.50	0.003	0.30
P10	MW	*F*(1,15) = 35.42	< 0.001	0.70
	MW x Freq.	*F*(3,45) = 15.52	< 0.0001	0.51
P11	MW	*F*(1,15) = 162.52	0.002	0.92
	MW x Freq.	*F*(3,45) = 39.53	< 0.0001	0.72
P12	MW	*F*(1,14) = 24.88	< 0.0001	0.64
	MW x Freq.	*F*(3,42) = 20.60	< 0.0001	0.60
P13	MW	*F*(1,15) = 12.49	0.015	0.45
	MW x Freq.	*F*(3,45) = 18.37	< 0.0001	0.55
P14	MW	*F*(1,14) = 0.37	1.0	0.03
	MW x Freq.	*F*(3,42) = 24.64	< 0.0001	0.64
P15	MW	*F*(1,15) = 0.38	1.0	0.03
	MW x Freq.	*F*(3,45) = 18.76	< 0.0001	0.56

#### Common spatial pattern and classification performance

**Fig 5:**
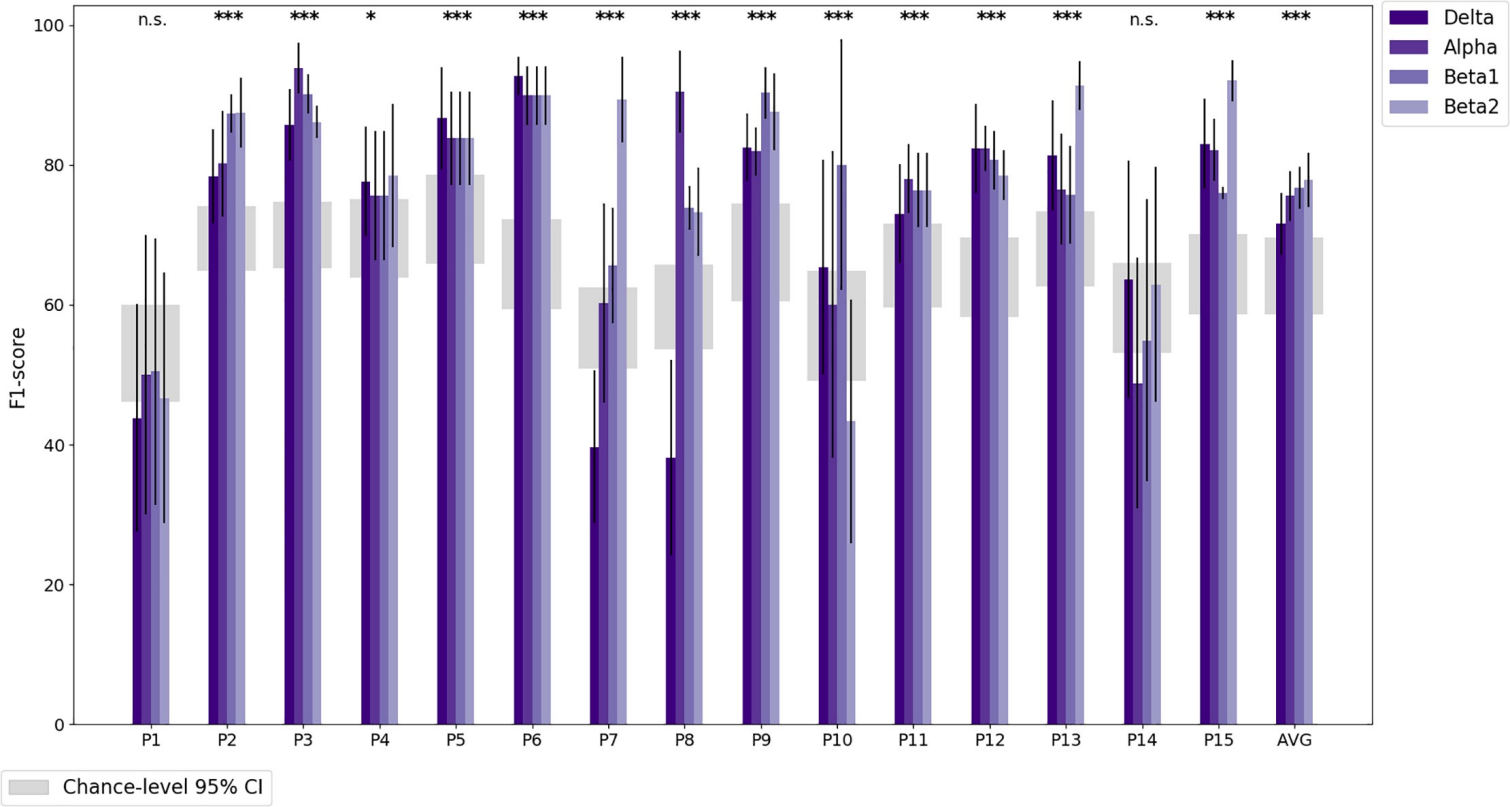
Classification performance for the Artifacts Suppressed approach given by the F1 score for each participant and frequency band. The rightmost set of bars are the averages across participants. Standard error bars are given for five cross-validation runs for each participant and all 15 participants for the averaged accuracies. The 95% confidence intervals for chance-level F1 scores per participant are plotted as grey regions, and statistical significance is calculated using independent two-sample t-tests using the best frequency band. *: *p* < 0.01, **: *p* < 0.001, ***: *p* < 0.0001.

**Fig 6:**
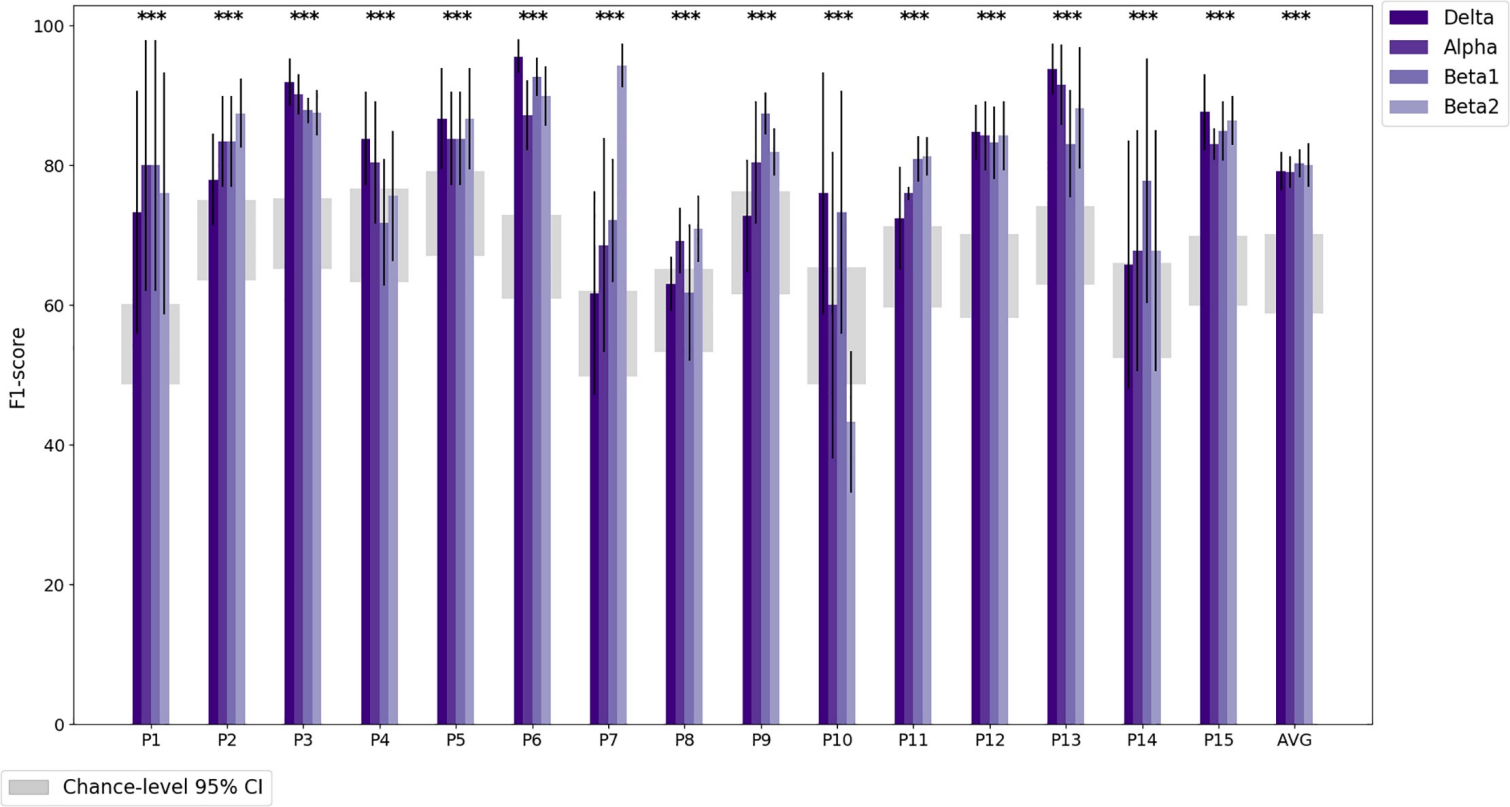
Classification performance for the Artifacts Present approach given by the F1 score for each participant and frequency band. The rightmost set of bars are the averages across participants. Standard error bars are given for five cross-validation runs for each participant and all 15 participants for the averaged accuracies. The 95% confidence intervals for chance-level F1 scores per participant are plotted as grey regions, and statistical significance is calculated using independent two-sample t-tests using the best frequency band. *: *p* < 0.01, **: *p* < 0.001, ***: *p* < 0.0001.

**Fig 7:**
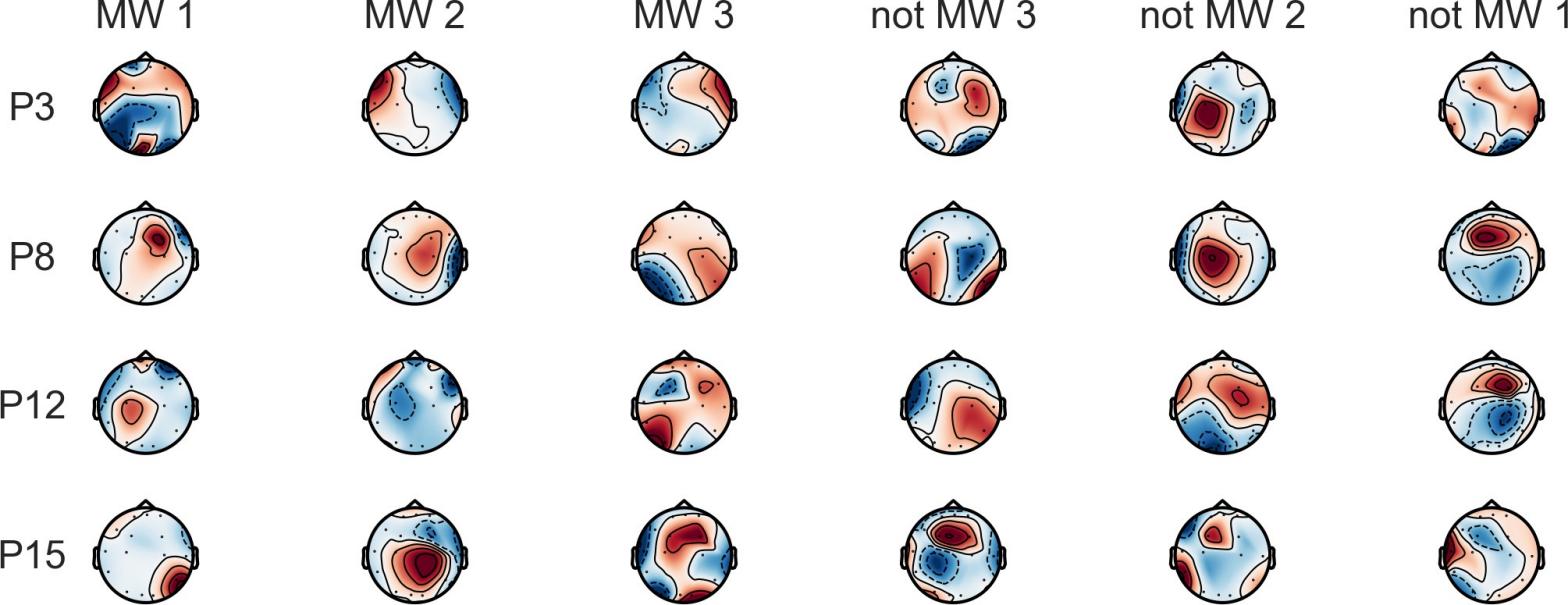
The learned common spatial pattern for four selected participants within frequency band yielding optimal detection (due to space restrictions) after artifact rejection. Note that these patterns do not reflect brain activations; rather, they show where the greatest *change in activation* took place between MW versus not MW. Pattern one refers to the learned pattern that is most strongly indicative of MW or not MW (listed as MW 1 or not MW 1), and increasing pattern number refers to less predictive patterns.

Classification performance in terms of the F1 score is shown in Fig 5 for MW detection for the Artifacts Suppressed approach and in Fig 6 for the Artifact Present approach. The average intra-subject classification performance was well above chance for both approaches (Artifacts Suppressed: *M* = 0.83, *SD* = 0.12; Artifacts Present: *M* = 0.85, *SD* = 0.07) when considering the best frequency band per individual. The classification performance for the best frequency band per individual is shown in Table 6 for the Artifacts Suppressed approach and Table 7 for Artifacts Present approach. Inter-subject classification did not yield classification performance above chance levels (Artifacts Suppressed best frequency band: *M* = 0.56, *SD* = 0.12; Artifacts Present: *M* = 0.56, *SD* = 0.11). In other words, we were able to predict MW in individuals, but the neural patterns of MW differed across participants, as shown in Fig 7.

**Table 6:** Classification performance (with standard error across cross-validation runs) for the best frequency band using the common spatial pattern algorithm with the Artifacts Suppressed approach. Frequency band chosen based on F1 score. Average performance (with standard deviation across participants) is given by the best frequency band for each participant. Observed MW rate reflects the MW rate of the processed epochs after epoch removal, and therefore may not be the exact rate of MW observed in the probe responses. Predicted MW rate is the rate at which the machine learning model predicts MW.

	Best Band	Accuracy	F1 Score	Recall	Precision	Observed MW Rate	Predicted MW Rate
P1	Beta1	88 ± 4.38	0.50 ± 0.19	0.45 ± 0.18	0.60 ± 0.22	0.53	0.80
P2	Beta2	80 ± 7.30	0.87 ± 0.05	1.00 ± 00.0	0.79 ± 0.07	0.25	0.03
P3	Alpha	90 ± 5.96	0.94 ± 0.04	1.00 ± 0.00	0.89 ± 0.06	0.25	0.10
P4	Beta2	70 ± 13.04	0.78 ± 0.10	1.00 ± 0.00	0.70 ± 0.13	0.25	0.00
P5	Theta	80 ± 10.95	0.87 ± 0.07	1.00 ± 0.00	0.80 ± 0.11	0.14	0.05
P6	Theta	88 ± 4.38	0.93 ± 0.03	1.00 ± 0.00	0.87 ± 0.05	0.31	0.16
P7	Beta2	90 ± 5.96	0.89 ± 0.06	0.83 ± 0.09	1.00 ± 0.00	0.50	0.60
P8	Alpha	92 ± 4.38	0.90 ± 0.06	0.90 ± 0.09	0.95 ± 0.04	0.44	0.40
P9	Beta1	85 ± 5.48	0.90 ± 0.04	1.00 ± 0.00	0.83 ± 0.06	0.29	0.15
P10	Beta1	100 ± 0.00	0.80 ± 0.17	0.80 ± 0.18	0.80 ± 0.18	0.50	0.47
P11	Alpha	67 ± 6.67	0.78 ± 0.05	1.00 ± 0.00	0.65 ± 0.07	0.33	0.03
P12	Alpha	73 ± 3.65	0.82 ± 0.03	0.97 ± 0.03	0.78 ± 0.09	0.36	0.10
P13	Beta2	90 ± 3.65	0.91 ± 0.04	1.00 ± 0.00	0.85 ± 0.06	0.27	0.23
P14	Theta	76 ± 8.76	0.64 ± 0.17	0.76 ± 0.17	0.61 ± 0.18	0.44	0.36
P15	Beta2	90 ± 3.65	0.92 ± 0.03	0.95 ± 0.04	0.91 ± 0.05	0.36	0.33
**Avg.**		**84 ± 8.96**	**0.83 ± 0.12**	**0.91 ± 0.15**	**0.80 ± 0.12**	**0.35 ± 0.11**	**0.25 ± 0.23**

**Table 7:** Classification performance (with standard error across cross-validation runs) for the best frequency band using the common spatial pattern algorithm with the Artifacts Present approach. Frequency band chosen based on F1 score. Average performance (with standard deviation across participants) is given by the best frequency band for each participant. Observed MW rate reflects the MW rate of the processed epochs after epoch removal, and therefore may not be the exact rate of MW observed in the probe responses. Predicted MW rate is the rate at which the machine learning model predicts MW.

	Best Band	Accuracy	F1 Score	Recall	Precision	Observed MW Rate	Predicted MW Rate
P1	Alpha	100 ± 0.00	0.80 ± 0.18	0.80 ± 0.18	0.8 ± 0.18	0.53	0.68
P2	Beta2	80 ± 7.30	0.87 ± 0.05	1.00 ± 0.00	0.79 ± 0.07	0.25	0.03
P3	Theta	87 ± 5.58	0.92 ± 0.03	1.00 ± 0.00	0.86 ± 0.06	0.25	0.07
P4	Theta	80 ± 8.37	0.83 ± 0.07	1.00 ± 0.00	0.75 ± 0.10	0.25	0.10
P5	Theta	80 ± 10.95	0.87 ± 0.07	1.00 ± 0.00	0.80 ± 0.11	0.14	0.05
P6	Theta	92 ± 4.38	0.96 ± 0.02	1.00 ± 0.00	0.92 ± 0.04	0.31	0.20
P7	Beta2	93 ± 3.65	0.94 ± 0.03	0.90 ± 0.05	1.00 ± 0.00	0.50	0.57
P8	Beta2	64 ± 6.69	0.71 ± 0.05	0.86 ± 0.09	0.65 ± 0.08	0.44	0.28
P9	Beta1	80 ± 4.47	0.87 ± 0.03	1.00 ± 0.00	0.78 ± 0.05	0.29	0.10
P10	Theta	87 ±7.30	0.76 ± 0.17	0.73 ± 0.17	0.80 ± 0.18	0.50	0.47
P11	Beta2	73 ± 3.65	0.81 ± 0.03	0.96 ± 0.04	0.72 ± 0.04	0.33	0.17
P12	Theta	80 ± 5.58	0.85 ± 0.04	0.89 ± 0.06	0.85 ± 0.08	0.36	0.23
P13	Theta	93 ± 3.65	0.94 ± 0.04	0.96 ± 0.04	0.93 ± 0.06	0.27	0.33
P14	Beta1	96 ± 3.58	0.78 ± 0.17	0.76 ± 0.17	0.80 ± 0.18	0.44	0.56
P15	Theta	87 ± 5.58	0.88 ± 0.05	0.85 ± 0.09	0.95 ± 0.04	0.36	0.43
**Average**		**85 ± 9.11**	**0.85 ± 0.07**	**0.91 ± 0.09**	**0.83 ± 0.09**	**0.35 ± 0.11**	**0.28 ± 0.20**

It is noteworthy that for some participants the Artifacts Present approach yielded the best classification performance, suggesting a potential role of ocular and some motor artifacts in MW detection. On average, the Artifacts Suppressed approach produced a slightly lower classification accuracy than the Artifacts Present, but this difference was not significant using a two-sided, paired-samples t-test, *t*(14) = -0.30, *p* = 0.77.

We computed Spearman’s ρ (rho) between the number of epochs available for training and the F1 score for each participant to check whether the variation in classification accuracy could partially be explained by the amount of available training data. For the Artifacts Suppressed classification accuracies, we found that ρ = 0.28, *p* = 0.32, indicating no significant relationship. For the Artifacts Present approach, we found that ρ = 0.51, *p* = 0.05, suggesting that there may be a relationship, and that additional training data may improve accuracy.

**Fig 8:**
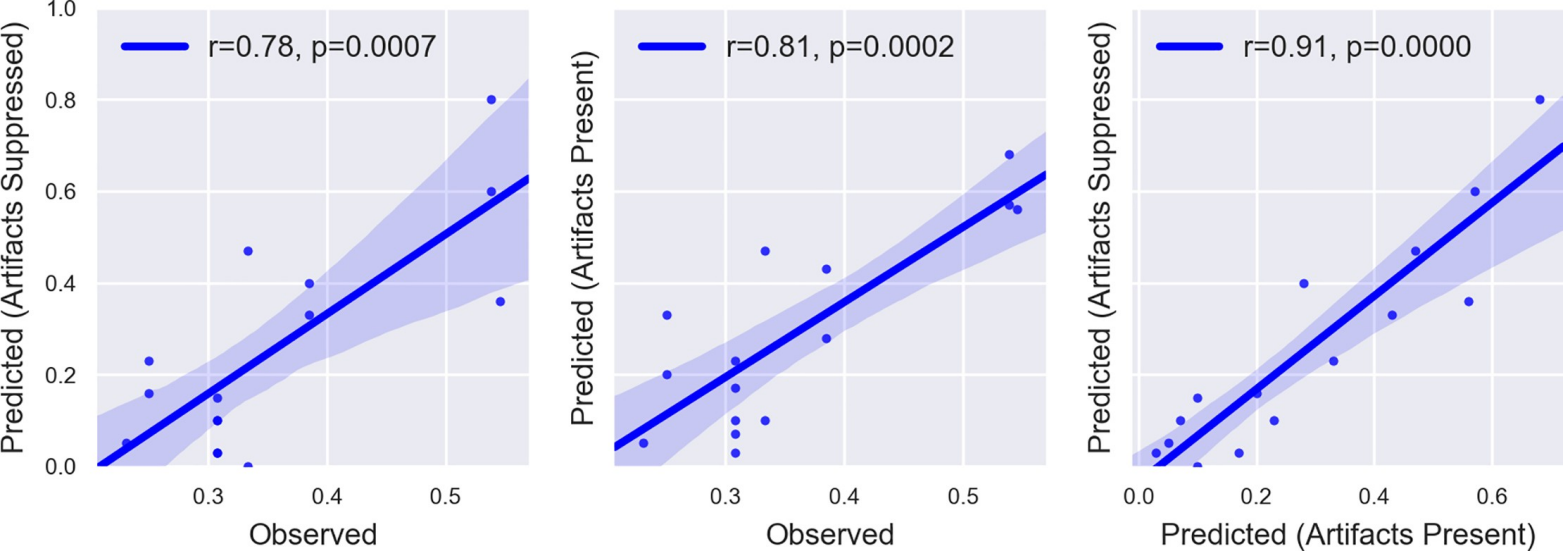
The relationships between observed and predicted MW rates.

In addition to measuring the classification accuracy of our models, we compared their predicted MW rates per participant with each participant’s actual MW rate (see Fig 8). For both the Artifacts Suppressed and Artifacts Present approaches, observed and predicted rates were highly correlated (Artifacts Suppressed: *r* = 0.78, *p* = 0.0007; Artifacts Present: *r* = 0.81, *p* = 0.0002). Additionally, the predicted MW rates obtained with both approaches were very highly correlated with one another (*r* = 0.91, *p* < 0.0001).

## Discussion

In this study, we used machine learning methods designed for data-driven feature learning with EEG to detect MW during live lectures at both the individual and group levels. While our data revealed some similarities in the neural correlates of MW that have been found in prior studies, the neural correlates found in our study were much more highly individualized than previously reported. Our work suggests that understanding MW and developing applications based on MW detection may considerably benefit from methods that are capable of single-subject analysis of brain activity. This work suggests that further research on the individual variability of MW using signal processing methods that are more suitable for interpreting which networks are activated during MW is needed.

### Behavioural findings

Consistent with the majority of the literature in the field, we found that MW occurred on average 35% of the time during the lectures based on participant responses to thought probes. However, individual rates of MW varied considerably, as can be seen in Fig 7, likely reflecting a combination of individual propensity to MW, interest in the lecture material, and circumstantial factors (e.g., how well each participant slept the night before the study). The considerable variability in MW rates further illustrates the need for individual-level analysis of MW as a means of understanding the phenomenon in more detail. The individual predicted rates of MW obtained by our machine learning approach varied considerably as well, but were not significantly different from the observed rates. Fig 8 shows that the predicted rates of MW were highly correlated with the observed rates, providing further evidence that the models were able to capture something useful about MW on an individual basis.

Similar MW rates were found using thought probes in a classroom-based study [88], but in that case, MW was also found to be predictive of lower academic performance [16]. Contrary to what might be expected, we did not find a significant correlation between the prevalence of MW and recall or retention. A likely explanation is that the quiz questions used in our study were sent to us by the lecturers in advance, and therefore not directly linked to the content that was presented immediately before the thought probes. There was no mechanism used to connect the quiz items to the thought probes because we did not want the lecturers to be any more aware of when the probes would appear than the students to lower the probability of accidental cueing. This means that we had no way of truly knowing if participants were mind wandering when testable content was covered. Trainee motivation to learn and retain the content may also have been a factor, as anecdotal feedback suggested that not all participants found the lecture content relevant to their day-to-day clinical experiences.

Our findings also indicate some differences in participants’ behaviours during the two lectures. While the level of MW was roughly consistent across the various time points sampled in Lecture 1, there were two time points during Lecture 2 at which participants were significantly more likely to report MW. Lecture 2 also had overall poorer immediate recall and retention than Lecture 1. While no specific changes in the lecturer’s behaviour were documented during the two time points previously mentioned, the observed effects may have at least in part been due to differences in participants’ interest in the lecture content (there was more variability in participants’ reactions to Lecture 2). They may also have been related to the order in which the lectures were presented. Since Lecture 2 took place after participants had been in the LIVELab for nearly two hours, participants may have started to become fatigued, and thus may have been less motivated to pay attention to or retain content.

As stated earlier, the retention score data for Lecture 2 did not meet the assumptions of normality. However, since the significant differences reported were relatively large, they are unlikely to be false positives. Additionally, the correlational analyses reported in our study were underpowered due to a small sample size and we were unable to link the quiz questions in our study directly to the probes. However, since participants tended to MW at different time points throughout the lectures, linking the quiz questions and probes may ultimately have been of limited benefit as there would likely not have been enough data points to determine if those who were paying attention at a particular time point performed better on the corresponding quiz questions than those who were not. Linking the two may also have inadvertently cued participants to better remember the content covered close to a probe.

### Mind wandering detection

We were able to classify MW significantly above-chance accuracy for most participants using only neuroelectric signals (the Artifacts Suppressed approach; see Fig 5). We were also interested in classification approaches that prioritized optimal MW detection over other considerations motivated by numerous studies showing that eye tracking was useful for MW detection [53–55]. For this reason, we also experimented with the Artifacts Present approach. Using this approach, we obtained significantly better than chance classification accuracy for all participants (see Fig 6). In particular, participants for whom MW was difficult to classify in the Artifacts Suppressed approach yielded results more in line with the other participants in the Artifacts Present approach. However, since classification accuracy was higher in the Artifacts Suppressed approach for some participants, the average classification accuracies were not significantly different between the two approaches.

Since our machine learning approach was based upon data-driven feature learning with common spatial patterns, a method adapted from brain-computer interfacing, we note a potential limitation in its application to MW detection. While the algorithm may learn spatial patterns that reflect different modes of MW (or perhaps mind wandering about different things) that may generalize well for an individual during a single session, the fact that it is a supervised algorithm means that its generalizability is limited to patterns already represented in the training data. Due to both the non-stationarity of brain activity and the day-to-day changes in mood, concerns, and preoccupations of an individual, it is possible that common spatial patterns learned on one day or in one context may not generalize to MW on other days or in other contexts. Indeed, this limitation of common spatial patterns is also present and actively being addressed in the brain-computer interfacing literature [89–91], and can be considered a limitation of supervised machine learning for non-stationary data more broadly [92].

Contrary to other studies identifying EEG correlates of MW using more traditional statistical analyses [40, 50], we did not find a consistent pattern of frequency band activation or spatial topographies that could be identified as a signature of MW across participants. While a recent study using machine learning to detect MW from EEG also showed that a variety of features of the EEG needed to be used together to reliably detect MW [18], our findings differed in that patterns of MW were highly individualized. In fact, our inter-subject model did not achieve above chance-level accuracy. The differences between our findings and those of previous studies can be explained by several factors, which we discuss next.

As discussed in the introduction, some of the discrepancies found in the neural correlates across EEG studies of MW may emerge from differences in experimental settings and the attentive task used as a control condition. As can be seen in Fig 3, our data were more in line with the EEG-derived neural correlates of MW discovered in more controlled laboratory-based experimental designs in which MW was contrasted against breath focus [40]. While we did not find an effect on the lower frequency range (i.e., theta), we did find a decrease in alpha power (which we defined as the more commonly used 8–12 Hz band, rather than 9–11 Hz) over occipital and parietocentral regions, although we note that these findings were only statistically significant before correcting for multiple comparisons. In addition, we found a similar decrease in beta power over frontolateral regions, with the addition of a decrease over the left occipital cortex. It is important to note that, unlike previous studies, we split our beta band into a lower beta band and a higher band and only found these significant effects in the higher frequency beta band. The main difference in our findings was a lack of effect in the theta band, which may be because our study took place in a more naturalistic setting with both auditory and visual stimuli. This may have resulted in a greater degree of ocular artifact contamination, which would have disproportionately affected lower frequency bands. In a study where the active task was listening to stories, a similar decrease in alpha power in occipital and parietal areas was found when participants were aware that they were thinking about something other than the task, and were still partially attending to the task [52]. In contrast, this study showed a widespread increase in alpha activity when participants were not aware that they were no longer paying attention to the task. If we were to interpret our results in the same way, it is possible that, on average, the participants in our study were often aware that they were thinking about something other than the lectures, and thus were intentionally mind wandering [93].

### Individual-level neural correlates of mind wandering

A seemingly unique finding in our study was the highly individualized nature of the neural correlates of MW that were found via data-driven feature learning (common spatial patterns), in combination with the inability of our machine learning approach to identify patterns that could generalize across participants. This can be seen in Fig 4, where MW was associated with changes in band powers in different directions for different participants, and in Fig 7, where the spatial patterns most predictive of MW only showed almost no similarity across individuals (although for participants P3, P8, and P12, for whom the alpha band is shown, there were similarities in the common spatial patterns associated with not MW).

The variety of neural correlates found across individuals is too broad to identify any consistent patterns that can be associated with the neural networks identified in previous studies using EEG [38]. Moreover, with 16 EEG channels, we lack the spatial resolution needed for accurate source localization [94, 95]. Furthermore, common spatial patterns only reflect scalp topologies that maximize the ratio of amplitude variance between two cognitive states, and may, therefore, omit brain regions that are activated in both states. As such, we would not necessarily expect a one-to-one correspondence between the common spatial patterns learned from the MW data and the network of brain regions that are associated with MW. With this limitation in mind, we discuss a possible interpretation of the common spatial patterns seen in Fig 7 to motivate further research exploring what machine learning methods may reveal about the neural correlates of MW, and other cognitive processes, based on individual-level analysis.

A study exploring the EEG scalp topologies (as opposed to common spatial patterns) in different frequency bands that appear during the activation of different resting-state networks in fMRI [96] may allow us to infer something about what the common spatial patterns reveal about the neural correlates of MW across individuals. Visually, the MW patterns for P3 are similar to the alpha EEG activity associated with visual networks, whereas the patterns associated with MW in P8, P12 and P15 are more reminiscent of the EEG activity associated with co-activation of the DMN, the frontoparietal control network, and the frontal attention network. All of these networks have been associated with MW through prior neuroimaging research [7]. For P3, P8, and P12, the most predictive common spatial patterns and the most similar network-related scalp topologies were found in the alpha band, activity in which has been specifically linked to resting state functional brain activity [96–100] and a decrease in bottom-up sensory processing [97, 101]. For P15, while the common spatial patterns appear distinct because they show changes in the beta2 frequency band instead of alpha, the most similar beta band scalp topology shown in [96] is associated with the same networks as P8 and P12. This suggests that for P15, a reduction in beta activity, which is associated with a decrease in active thinking and concentration [102], was more predictive than an increase in alpha activity, even though they may reflect a change in the same resting-state networks and suggest a very similar change in cognitive state.

Comparing the common spatial patterns discovered through individual-level feature learning to EEG scalp topologies associated with specific brain networks may help explain why group-level analyses of functional connectivity in fMRI data consistently point to the same networks, but different EEG studies at times appear to show contradictory neural correlates (see our review of the neural correlates of MW in the introduction). This interpretation would suggest that while MW may generally be associated with changing activity patterns in the same set of resting-state networks, the oscillatory dynamics of those networks may change in different ways for different individuals, and different subnetworks may be activated at different times. This leads to a very interesting hypothesis about the dynamics and varieties of MW that warrants further exploration. However, we caution readers against over-interpreting these findings by assuming that common spatial patterns necessarily reveal the same brain networks discovered in previous studies. While similar inferences about the network activations for each participant can be drawn by comparing the learned common spatial patterns to scalp topologies identified in simultaneous EEG-fMRI studies, doing so in a rigorous way requires an entirely different kind of experimental approach that is outside of the scope of this paper. Ideally, the association between individual-level common spatial patterns and network activations would be tested by computing common spatial patterns from EEG data that were simultaneously acquired with fMRI.

We note two reasons for finding patterns of brain activity associated with MW that are more individualized than previously reported. The first is that the common spatial patterns algorithm may be particularly well-suited to machine learning analysis on an individual basis, as its roots are found in within-subject brain-computer interfacing research [85, 86]. Common spatial patterns may, therefore, identify patterns that are highly tuned to individuals and can be especially powerful in identifying patterns that would likely be lost upon averaging across individuals, or in other group-level analyses. The tradeoff is that this method may not be well-suited to generalization across individuals, and as noted earlier, recent research in brain-computer interfacing has focused on developing new methods specifically designed to overcome this limitation [103, 104].

Second, our broad content-based definition of MW may have led to a large degree of heterogeneity in the neural correlates of MW across participants, particularly if there was variation in how the thought probes were interpreted [25]. This may have further contributed to poor inter-subject generalizability in our machine learning models. We explained earlier that we chose to use a broad definition of MW so that we could discover neural correlates of MW that were more likely to translate well into real-world applications related to our naturalistic setting (i.e., MW detection and/or attentional monitoring of trainees during live lectures). High variation has been found in previous work, and is thought to reflect differences in the content of thought during MW and while paying attention [93, 105], which can, in turn, lead to the activation of different neural networks in the brain [106]. We add support to this hypothesis using traditional statistical analyses. As can be seen in Figs 3 and 4, almost every participant showed differences between MW and non-MW EEG epochs in multiple frequency bands. However, after combining the epochs across participants and comparing MW to non-MW EEG at each EEG channel, the differences almost entirely disappeared, suggesting that these differences did not generalize across participants. By showing that machine learning and data-driven feature learning can be used to detect MW on an individual basis, we can contribute further evidence that MW could be considered a highly variable phenomenon that can be expressed broadly across the neocortex and across a wide range of frequencies.

The application of machine learning for predictive modelling in other areas of neuroscience has revealed the possibility of neural correlates of various mental processes that were not previously identified in group-level statistical analyses [62, 64–69, 85, 86]. It is possible that individual-level analyses performed on fMRI data may also reveal that MW involves a greater variety of neural correlates and brain networks than could be identified through group-level analyses. Such individual analyses of MW may contribute substantially towards resolving the question of whether these different patterns of neural activation correspond to different types (i.e., intentional versus unintentional [93, 107]) or definitions of (i.e., content-based versus stimulus-independent versus spontaneous thought [7]) of MW, and furthermore, if it is possible to differentially detect types of neural activity unrelated to the task at hand. Future work could use simultaneous EEG-fMRI with individual-level modelling to gain a more precise understanding of the individual variability in network activation involved in MW, including how those networks interact. In addition, such data could be used to clarify the relationship between machine learning derived common spatial patterns that enable MW detection by establishing the correlation between the appearance of those patterns and specific brain networks. This may help resolve whether the executive control failure hypothesis or the decoupling hypothesis is closer to reality, or if neither is a sufficient description of why the executive control network can be co-active with the DMN during MW.

## Conclusions

We were able to accurately detect MW from EEG at the individual level using data-driven feature learning and machine learning. These methods allowed us to show that the neural correlates of MW might be more variable than suggested by traditional statistical methods. With further study, these findings may lead to the development of new methods for online MW detection that facilitate the deeper study of the phenomenon, particularly at the individual level, while also enabling real-time MW detection in real-world settings. This work points to the possibility that MW might be associated with multiple patterns of activity in previously identified resting state brain networks, which are best revealed by analysis of brain activity at the individual level.

## Supporting information

S1 Appendix Lecture 1 (intimate partner violence) quizzes. (DOCX)

S2 Appendix Lecture 2 (meta-analytic methods in orthopedic research) quizzes. (DOCX)
