# Supplementary material for: Individualized pattern recognition for detecting mind wandering from EEG during live lectures
Source: PLoS One. 2019 Sep 12;14(9):e0222276. doi: 10.1371/journal.pone.0222276 (PMC6742406; doi:10.1371/journal.pone.0222276)
Supplement: S1 Appendix — (DOCX) [file pone.0222276.s001.docx]

**S1 Appendix. Lecture 1 (intimate partner violence) quizzes.**

Immediate recall:

1. How interesting did you find the content of this presentation?
   1. Very interesting
   2. Interesting
   3. Neither interesting or uninteresting
   4. Uninteresting
   5. Very uninteresting
   6. I don’t know
2. How engaging did you find the presenter?
   1. Very interesting
   2. Interesting
   3. Neither interesting or uninteresting
   4. Uninteresting
   5. Very uninteresting
   6. I don’t know
3. Approximately 1 in ___ women experience IPV in their lifetime according to the PRAISE-2 pilot study.
4. What is the best-reported IPV study design?
5. A central theme of the presentation is that research quality is linked with what concept?
6. Name one of the two largest clinical trial registries identified by the presenter.

Retention:

1. The Cochrane review by Doherty et al. was a high-quality systematic review of IPV screening studies. However, the presenter raised several issues with the studies in the review. Briefly identify one issue raised by the presenter.
2. Failure to publish studies, especially based on how interesting or newsworthy they are, can result in what type of bias?
3. As opposed to conventional journal citations, alternative metrics measure what type of mentions?
4. Identify one way that pre-print servers are different from traditional journals, according to the presenter.
5. What study design is PRAISE-2?
