# Supplementary material for: Individualized pattern recognition for detecting mind wandering from EEG during live lectures
Source: PLoS One. 2019 Sep 12;14(9):e0222276. doi: 10.1371/journal.pone.0222276 (PMC6742406; doi:10.1371/journal.pone.0222276)
Supplement: S2 Appendix — (DOCX) [file pone.0222276.s002.docx]

**S2 Appendix. Lecture 2 (meta-analytic methods in orthopedic research) quizzes.**Immediate recall:

1. How interesting did you find the content of this presentation?
   1. Very interesting
   2. Interesting
   3. Neither interesting or uninteresting
   4. Uninteresting
   5. Very uninteresting
   6. I don’t know
2. How engaging did you find the presenter?
   1. Very interesting
   2. Interesting
   3. Neither interesting or uninteresting
   4. Uninteresting
   5. Very uninteresting
   6. I don’t know
3. The key differentiator between pairwise and network meta-analysis is the ability to compare _____________ interventions within the network.
4. Within a network meta-analysis, assessing the effect between two treatments through a common operator is called an _____________ effect.
5. What is the term used to describe when direct and indirect effects do not agree with one another?
6. What type of analysis is used to model the rankings of all included treatments?
7. What piece of information does treatment ranking within a network meta-analysis not provide?

Retention:

1. What is the name of the assumption required for network meta-analysis in which there are no systematic differences between comparisons across the network?
2. What is the term used to describe the treatment effect created through combining the direct and indirect effects between two treatments?
3. What term is used to describe a network with few trials and/or patients included within it?
4. What type of score is used to rank treatments based on probabilities of being the best treatment option?
5. What is a current difficulty regarding the reporting of network meta-analysis results that our team has proposed to improve?
